# Effects of the Monomeric Components of Poly-hydroxybutyrate-co-hydroxyhexanoate on the Growth of *Vibrio penaeicida* In Vitro and on the Survival of Infected Kuruma Shrimp (*Marsupenaeus japonicus*)

**DOI:** 10.3390/ani11020567

**Published:** 2021-02-22

**Authors:** Kimio Fukami, Fumika Takagi, Kohei Sonoda, Hiroshi Okamoto, Daisuke Kaneno, Takao Horikawa, Masaki Takita

**Affiliations:** 1 Faculty of Agriculture and Marine Science, Kochi University, Nankoku-Shi, Kochi 783-8502, Japan; takagifumika1026@gmail.com (F.T.); kaneno@kochi-u.ac.jp (D.K.); 2 HIGAHIMARU CO., Ltd. Ichikikushikino-Shi, Kagoshima 896-0046, Japan; k-sonoda@k-higashimaru.co.jp (K.S.); h-okamoto@k-higashimaru.co.jp (H.O.); 3 Kaneka Corporation, Nakanoshima, Osaka 530-8288, Japan; Takao.Horikawa@kaneka.co.jp (T.H.); Masaki.Takita@kaneka.co.jp (M.T.)

**Keywords:** poly-hydroxybutyrate-co-hydroxyhexanoate (PHBH), short- and medium-chain hydroxyalkanoic acids, pathogenic *Vibrio*, inhibitory effect, shrimp survival, *Marsupenaeus japonicus*

## Abstract

**Simple Summary:**

Outbreaks of bacterial disease in shrimp aquaculture are major causes of economic and production losses. It is, therefore, important to prevent such outbreaks, preferably without the use of antibiotics to avoid encouraging the development of antibiotic-resistant bacterial strains. Several hydroxyalkanoic acids are known to inhibit the growth of pathogenic *Vibrio* species. Poly-hydroxybutyrate-co-hydroxyhexanoate is a biodegradable, water-insoluble polymer comprising two hydroxyalkanoic acids: 3-hydroxybutyrate and 3-hydroxyhexanoate. Here, we evaluated the inhibitory activities of 3-hydroxybutyrate and 3-hydroxyhexanoate on the growth of the shrimp-pathogenic bacterium *Vibrio penaeicida* in vitro, and the effect of adding poly-hydroxybutyrate-co-hydroxyhexanoate to the diet on the survival of infected kuruma shrimp (*Marsupenaeus japonicus*). We found that the activities of 3-hydroxybutyrate and 3-hydroxyhexanoate were pH dependent and that they inhibited bacterial growth at pH close to that found in the shrimp gut (pH 5.9–6.7). Moreover, in shrimp infected with *V. penaeicida*, survival rates were significantly increased in individuals reared on feed containing poly-hydroxybutyrate-co-hydroxyhexanoate compared with those reared on standard diet, without any negative effects on shrimp growth. These findings suggest that supplementation of standard diet with poly-hydroxybutyrate-co-hydroxyhexanoate could protect aquaculture shrimp from infection by *V. penaeicida*, which is expected to increase production and reduce overall operational costs.

**Abstract:**

Here, we investigated the inhibitory effects of the biodegradable, water-insoluble polymer poly-hydroxybutyrate-co-hydroxyhexanoate (PHBH) and its two constituent monomers, the hydroxyalkanoic acids 3-hydroxybutyrate (3HB) and 3-hydroxyhexanoate (3HH), on the growth of the shrimp-pathogenic bacterium *Vibrio penaeicida*. In vitro experiments revealed that 3HH showed greater growth inhibitory activity than 3HB against *V. penaeicida*. In addition, the activities of hydroxyalkanoic acids were pH dependent, being greater at pH 6.0 than at pH 7.0. Investigation of the pH of the shrimp gut revealed a pH range of 5.9–6.7 (mean 6.29 ± SD 0.20), indicating that the physiological environment was suitable for 3HB and 3HH to exert their inhibitory activities against *V. penaeicida*. In vivo bacterial challenge experiments revealed that survival rates in kuruma shrimp (*Marsupenaeus japonicus*) infected by *V. penaeicida* were significantly increased in shrimp reared on feed containing PHBH (0.1%–5% *w*/*w* PHBH) compared with that in shrimp reared on standard diet alone. Supplementation with PHBH had no significant effects on three shrimp growth parameters: increase in body weight, daily feeding rate, and feed conversion ratio. These results suggest that supplementation of standard diet with PHBH will increase shrimp resistance to infection by *V. penaeicida*, thereby increasing shrimp aquaculture productivity.

## 1. Introduction

In 2018, aquaculture farming accounted for 61% (9387 × 10^3^ t) of the global Crustacean supply [1]. Shrimp production occupies a major part in Crustacean aquaculture and is performed mostly in China and several Southeast Asian countries [1]. In these countries, however, as shrimps are usually cultured at high density using commercial pellet feeds that contain high concentrations of nutrients, outbreaks of disease are common due to deterioration of water and sediment quality in the culture ponds [2,3,4,5]. In Japan, *Marsupenaeus japonicus* (kuruma shrimp) is a popular aquaculture species, with more than 300 million juveniles produced every year [6]. In 2018, around 1500 t of *M. japonicus* was produced by aquaculture [7] and serious outbreaks of diseases have often been reported in Japan [6,8]. Disease outbreaks in shrimp aquaculture are major causes of economic and production losses [9]. Among the various causes of these diseases, shrimp-pathogenic bacteria of the genus *Vibrio* are the most economically damaging, resulting in multibillion-dollar losses to the aquaculture industry [10,11]. Antibiotics are intensively used to control these bacteria [12,13,14]; however, this has resulted in the development of antibiotic-resistant bacterial strains [15,16]. Thus, methods for protecting aquaculture shrimps without the use of antibiotics are needed [17].

Short-chain fatty acids (FAs) have antibacterial activities and have been shown to inhibit the growth of pathogenic *V. campbellii* [18]. It has also been reported that the growth inhibitory activities of these FAs increase with increasing carbon number [18]. Moreover, FAs have been shown to significantly increase survival rate in *Artemia franciscana* (brine shrimp) nauplii challenged with *V. campbellii* [18]. However, short- and medium-chain FAs are usually water-soluble and so are diluted in the aquatic environment. Therefore, because high concentrations are needed to obtain effective doses, short- and medium-chain FAs are not economically attractive for disease control, in particular in large-scale aquaculture ponds [19]. To address this issue, Defoirdt’s group examined the use of water-insoluble polymers for bacterial control [20]. Poly-β-hydroxybutyrate (PHB), a water-insoluble polymer of the short-chain hydroxyalkanoic acid 3-hydroxybutyrate (3HB), is a carbon and energy storage compound accumulated by a wide variety of bacterial strains under conditions of nitrogen depletion and carbon excess [17,21]. Adding PHB or PHB-accumulating bacterial cells directly to the diet or rearing water has improved the survival rates of various aquaculture animal species such as Chinese mitten crab, *Eriocheir sinensis*, larvae [22], *Penaeus monodon* postlarvae [23], giant freshwater shrimp larvae [24], mussel larvae [25], Nile tilapia *Oreochromis niloticus* juveniles [26], and *Litopenaeus vannamei* postlarvae [27]. Defoirdt et al. [20] speculated that PHB was at least partially degraded to 3HB in the animal intestinal tract, and that it was the 3HB monomer that protected these aquatic animals from infection by *V. campbellii*. They also reported that 3HB inhibited the growth of *V. campbellii* in vitro although 3HB (β-hydroxybutyrate) showed less inhibitory effect than that of butyrate [20]. Defoirdt et al. [28], therefore, subsequently reported that high concentrations are needed for 3HB to exert its protective effects in *A. franciscana*.

In the 1990s, it was discovered that another biodegradable polymer, poly-hydroxybutyrate-co-hydroxyhexanoate (PHBH), was produced by select bacteria, including *Aeromonas caviae* [29,30] and a recombinant strain of *Cupriavidus necator* [31,32]. This intracellular PHBH is now commercially available as a purified powder. PHBH comprises two different hydroxyalkanoic acids: 3HB and 3-hydroxyhexanoate (3HH). Because the number of carbons in 3HH (C = 6) is greater than that in 3HB (C = 4), it can be expected that PHBH (a polymer of both 3HB and 3HH) would show greater inhibitory activity than PHB (a polymer of 3HB alone) on the growth of pathogenic *Vibrio* species [18].

Several previous studies have also shown that the inhibitory activities of short-chain FAs on the growth of *V. campbellii* are pH dependent [18,28,33,34]. For example, the growth of *V. campbellii* was markedly inhibited by 3HB (125 mM) at pH 6.0 but not at pH 7.0 [28]. This suggests that the pH of the shrimp gut could have a large impact on the activities of FAs. However, limited information is available on the pH of the shrimp gut.

Here, we developed a method for determining the pH of the shrimp gut and examined the growth inhibitory effects of 3HB and 3HH on the shrimp-pathogenic bacterium *Vibrio penaeicida* at physiologically relevant pH. We also examined the effects of supplementing standard diet with PHBH on shrimp growth and on survival after infection with *V. penaeicida*. We tried to connect inhibitory effects of monomeric components (3HB and 3HH) of PHBH (in vitro) and shrimp growth and survival due to PHBH degradation in shrimp gut (in vivo). Our findings suggest the possibility of using PHBH as a dietary supplement for use in shrimp aquaculture.

## 2. Materials and Methods

### 2.1. Pathogenic Bacterium and Growth Conditions

The shrimp-pathogenic bacterium *V. penaeicida* was isolated from a shrimp farm of the Higashimaru Corporation (Ichiki-Kushikino, Kagoshima Prefecture, Japan) in May 2013. The bacterial strain was cultured in FeTY nutrient medium [35] at 20 °C for 24 h. After preculturing twice, the cell suspensions were used for the subsequent experiments.

### 2.2. Preparation of Hydroxyalkanoic Acids and Fatty Acids

3HB was obtained from the AdipoGen Corporation (San Diego, CA, USA). Because 3HH was not commercially available, we prepared it ourselves as follows. Ethyl 3-hydroxyhexanoate (11.21 g, 70.0 mmol) was added to 100 mL of 5 M NaOH and stirred at room temperature for 4 h. The mixture was poured into a saturated aqueous NH_4_Cl solution and extracted with Et_2_O (3 × 100 mL). The combined extracts were dried over anhydrous Na_2_SO_4_ and filtered. After removal of the solvent under reduced pressure, pure 3HH was obtained as a colorless oil. The synthetic procedure described above was the most standard ester hydrolysis, and 1H NMR spectral analysis confirmed that the product was pure 3HH.

Because it is reported that the growth inhibitory activity of β-hydroxybutyrate is less than that of butyrate [20], the growth inhibitory activities of butyrate (C = 4) and hexanoate (C = 6), two fatty acids with the same carbon numbers as 3HB and 3HH, were also examined to provide data for comparison. Butyrate was obtained from the Tokyo Chemical Industry Corporation (Tokyo, Japan). Hexanoate was obtained from FUJIFILM Wako Pure Chemical Corporation (Osaka, Japan).

### 2.3. pH of the Shrimp Gut

Since no readily available equipment or technical solution for determination of shrimp gut pH existed, we developed it by ourselves as follows: frozen shrimp were thawed in room temperature within one hour, and the gut was removed using needles and placed in an Eppendorf tube (1.5 mL). An aliquot (0.2 mL) of sterilized seawater was added to the tube, the gut was homogenized by hand with a glass rod, and the pH of the homogenate was measured with pH test paper (EMD Millipore Corporation, Darmstadt, Germany). Then, another aliquot of seawater was added to the homogenate, and the pH was again determined after mixing. This process was repeated until a total of 1 mL of seawater had been added to the homogenate. Finally, the pH values were plotted against the amount of seawater added, and a line of best fit was added to the data. The approximate pH values in the shrimp gut were then estimated by extrapolation back to the *y*-axis.

Here, two experiments were conducted. In the first, two shrimps reared on standard diet and two shrimps reared on feed supplemented with 5% (*w*/*w*) PHBH were used after the end of the 6-week feeding experiment (see Section 2.5). In the second, 20 shrimps of various sizes reared on standard diet were used.

### 2.4. Growth Inhibitory Effects in V. penaeicida

3HB, 3HH, butyrate, or hexanoate was added to FeTY culture medium to a final concentration of 0 (control), 10, 50, or 100 mM, and the pH of the medium was adjusted to 6.0 or 7.0 using 1N-NaOH or 1N-HCl solutions. The medium (10 mL for 3HB and 3HH, and 100 mL for butyrate and hexanoate) was then inoculated with a 0.01 or 0.1 mL of the cell suspension of *V. penaeicida* (with an estimated cell density of about 1 × 10^8^ cells/mL), prepared as described previously (see Section 2.1), and incubated at 20 °C for two days. Subsamples of the culture medium (0.1 mL for 3HB and 3HH, and 1 mL for butyrate and hexanoate) were collected at 24 h and 48 h after the start of incubation. After serial one-tenth dilutions of the cell suspension, 0.1 mL of each dilution was spread on FeTY agar plates in six replicates. After incubation for two days at 20 °C, the number of colonies on the plates were counted and the viable counts of the bacterium were obtained as the average of 6 plates.

### 2.5. Shrimp Growth Experiments

Into 200-L water tanks containing sand to a depth of 2 cm, 100 L of seawater was added. Shrimp growth experiment was conducted for six weeks in duplicate tanks of five test sets (see below) each containing 30 individuals. Average weight of total 30 individuals for each tank (*n* = 10) was 37.6 ± 0.05 g (1.25 g for one individual). The water temperature of the rearing tanks during the experiment was 26.4–28.6 (average 27.4) °C. The seawater was constantly refreshed (1 rotation/h).

Purified, powdered PHBH (3HB:3HH = 89:11 as mol ratio) produced by a recombinant strain of *C. necator* (Kaneka Corporation, Hyogo, Japan) [32] was mixed with commercially available standard diet (Vitalprawn, Higashimaru Corporation, Ichiki-Kushikino, Kagoshima Pref., Japan). Ingredient compositions of the standard diet is shown in Table 1. Main sources of the standard diet are fish meal, squid meal, krill meal, and gluten. After smashing these materials by a grinding machine, trace minerals and oil, in addition to water, were added and mixed. All ingredients were extruded to make pellets with the size of 3 × 6 mm. Finally, pellets prepared were dried using a special drying machine until the water content was less than 10 %. Five different feeds were used: 0% PHBH (control); 0.1%, 1%, and 5% (*w*/*w*) PHBH (added 0.1%, 1%, 5% of PHBH to the standard diet, respectively); and internal 5% (*w*/*w*) PHBH (in which 5% of the standard diet was replaced by PHBH). PHBH of each % was mixed before preparing feed pellets. Formulated compositions of the experimental feed are shown in Table 2.

Each test feed was given to the shrimps once a day in the evening until satiation. When uneaten residual feed remained until next morning, it was collected, and the weight was determined. Amount of feed consumed was obtained by subtracting the weight of residual feed from that of feed given in a previous day. When there was no residual feed, we increased appropriately the amount of feed to shrimps in the following day.

Every two weeks from the start of the experiment, all the shrimps in each tank were collected, and the numbers of surviving individuals were counted. After removing excess water around the shrimp body with a paper towel, the total body weight of all the shrimps excluding the dead individuals in each tank was also determined. Daily feeding rate (%) was calculated as follows:

Daily feeding rate (%/d) = amount of feed consumed (g) × (average body weight (g) × average number of rearing shrimps)^−1^ × (rearing days)^−1^ × 100 (1)

where the average body weight was calculated expediently as the mean value of the initial and final total body weights, and the average number of rearing shrimps was obtained as the mean value of the initial and final number of shrimps. The feed conversion ratio was calculated as the total dry weight (g) of feed consumed divided by the total wet weight increase (g) of the shrimps multiplied by 100 (%).

### 2.6. In Vivo Pathogenic Bacterial Challenge Experiments

In vivo bacterial challenge experiment was carried out basically using a standard method [36]. It was conducted for seven days following the growth experiment. After rearing for nine weeks, 14 individuals selected randomly from each test group (0%, outer 0.1%, 1%, 5%, and internal 5% PHBH) were used. Average body weight of all shrimps was 8.67g and there was no significant difference among each test group.

One liter of marine broth culture medium was inoculated with a stock culture of *V. penaeicida* and incubated at 28 °C for 16 h until a cell density of 5.0 × 10^7^ cfu/mL. Then, 400 mL of the bacterial culture solution was placed in a 100-L water tank and sand-filtered seawater was added to a total volume of 20 L to afford a cell density of *V. penaeicida* of about 1.0 × 10^6^ cfu/mL.

Shrimps kept in a net cage (W 22 cm × D 20 cm × H 5 cm) prior to the challenge experiments were submerged in the bacterial cell suspension tanks for 30 min to allow bacterial infection. During infection, the tanks were continuously aerated, and the water temperature was around 25 °C. After infection, the shrimps were transferred to a 65-L glass tank (W 60 cm × D 36 cm × H 30 cm) containing 45 L of sand-filtered natural seawater and reared under continuously aerated conditions. The water temperature was maintained at 25 °C using a bar heater. Half the total volume of rearing water was changed every 24 h. Each experimental feed (0, 0.1, 1, 5%, and internal 5%) similar to the growth experiment was given to the shrimp once a day until satiation during the rearing period. The numbers of living shrimp in each test group were recorded each day for seven days.

After the bacterial challenge experiment, lymphoid organs and hearts of all shrimps (surviving shrimps, *n* = 31, dead shrimps, *n* = 39) were aseptically picked up from each individual and streaked on plates of marine agar medium 2216 (BD Difco, NJ, USA) using a forceps. The plates were then incubated at 28 °C for 24 h. After incubation, isolated colonies were inoculated on plates of TCBS agar medium (Nissui Seiyaku, Tokyo, Japan) and identified as *V. penaeicida* by the colony color and a slide agglutination test anti-*V. penaeicida* serum (Itami, personal communication, Miyazaki University, Japan).

### 2.7. Statistics

Differences among the pH values of the shrimp gut, growth inhibitory effects in *V. penaeicida*, and shrimp growth experiments were examined by using Student’s *t*-test. Differences in shrimp survival in the pathogenic bacterial challenge experiment were examined by using the chi-square test. Differences were considered significant at *p* < 0.05.

## 3. Results

### 3.1. pH of the Shrimp Gut

**Figure 1:**
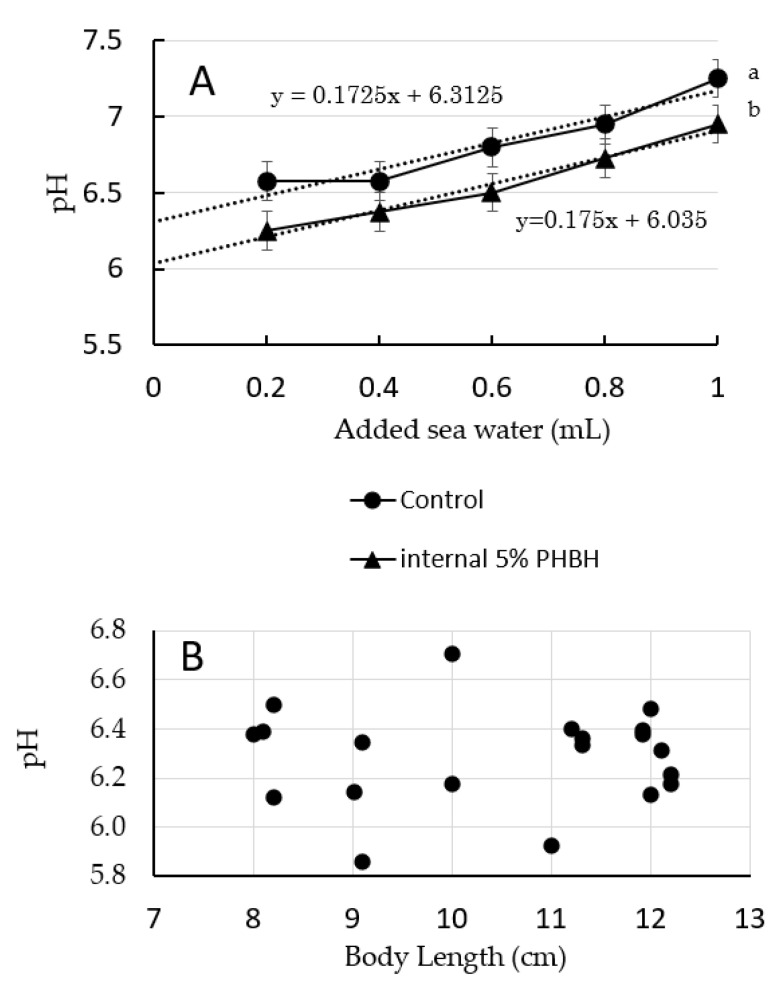
(**A**) Changes in pH of homogenized gut of *Marsupenaeus japonicus* versus the amount of seawater added to the homogenate. Shrimps were sacrificed after being reared for 6 weeks on standard diet (control) or experimental feed containing 5% (*w*/*w*) poly-hydroxybutyrate-co-hydroxyhexanoate (PHBH). Different superscript letters show significant differences (*p* < 0.05). The pH of the gut was estimated by extrapolating back to the *y*-axis of the best fit line. (**B**) pH values of the gut of *M. japonicus* individuals with different body lengths and reared on standard diet. pH values were obtained by the method shown in (**A**).

The pH of the guts of shrimp reared on feed with and without PHBH are shown in Figure 1A; average data from two individuals per diet are shown. The average gut pH of the shrimp reared on standard diet was around 6.3, whereas that of the shrimp reared on feed containing 5% PHBH was about 6.0. Although only two individuals were tested for each feed, the two averages were statistically different, suggesting that shrimps reared on feed containing 5% PHBH have a lower gut pH compared with that of shrimp reared on standard diet.

The pH of the guts of shrimp reared on standard diet are shown in Figure 1B plotted against body length. The pH of the guts of shrimp with body lengths of around 8–12 cm (average, 10.4 cm) (body weights about 6–19 g, average 14 g) was in the range of 5.9–6.7 (average 6.29 ± SD 0.20). No clear trend in gut pH by body length was observed.

### 3.2. Effects of Hydroxyalkanoic Acids and Fatty Acids on the Growth of V. penaeicida

**Figure 2:**
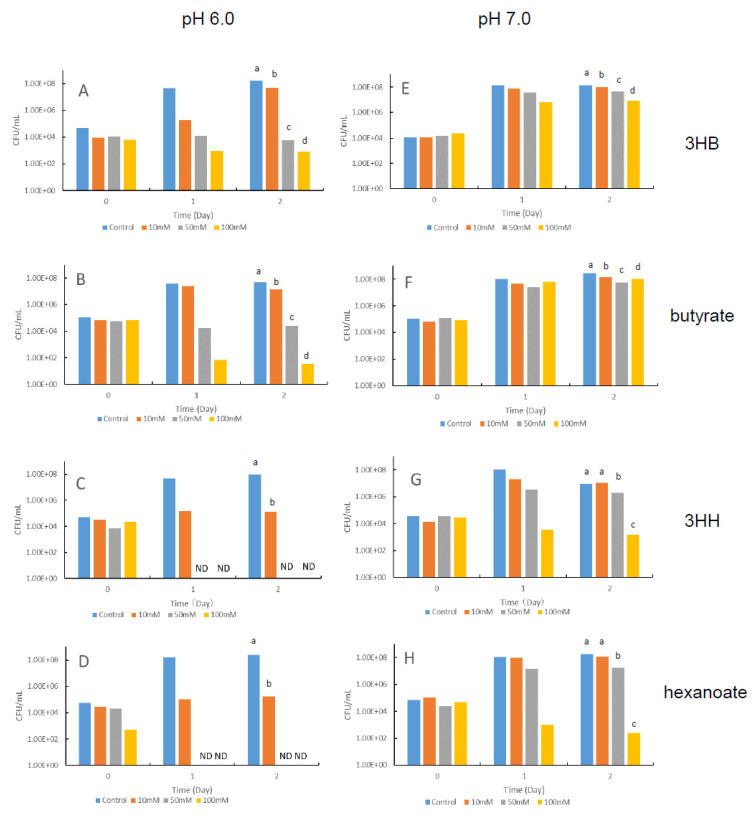
Growth of the shrimp-pathogenic bacterium *Vibrio penaeicida* in nutrient medium containing different concentrations of the hydroxyalkanoic acids 3-hydroxybutyrate (3HB) (**A**,**E**) or 3-hydroxyhexanoate (3HH) (**C**,**G**), or the fatty acids butyrate (**B**,**F**) or hexanoate (**D**,**H**) at pH 6.0 (left half) or 7.0 (right half). CFU: colony-forming units; ND: not detected. Different superscript letters in each figure show significant differences (*p* < 0.05).

First, we examined the pH dependence of the growth suppression activities of 3HB, 3HH, butyrate, and hexanoate against *V. penaeicida* in vitro (Figure 2). The data obtained from the experimental replicates were comparable; therefore, representative data are shown in Figure 2.

Overall, 3HH and hexanoate showed greater growth suppression activity than 3HB and butyrate, irrespective of pH, and greater growth suppression was observed at pH 6.0 than at pH 7.0 for all four compounds. At pH 6.0, greater growth suppression was observed with increasing concentration of 3HB, 3HH, butyrate, and hexanoate. 3HB and butyrate at 100 mM markedly suppressed growth compared with the control (Figure 2A,B), and 3HH and hexanoate completely stopped growth at 50 mM and 100 mM (Figure 2C,D). In contrast, at pH 7.0, no growth suppression was observed for 3HB and butyrate (Figure 2E,F), and 3HH and hexanoate inhibited growth only at 100 mM (Figure 2G,H).

### 3.3. Effect of PHBH on the Growth of M. japonicus

**Figure 3:**
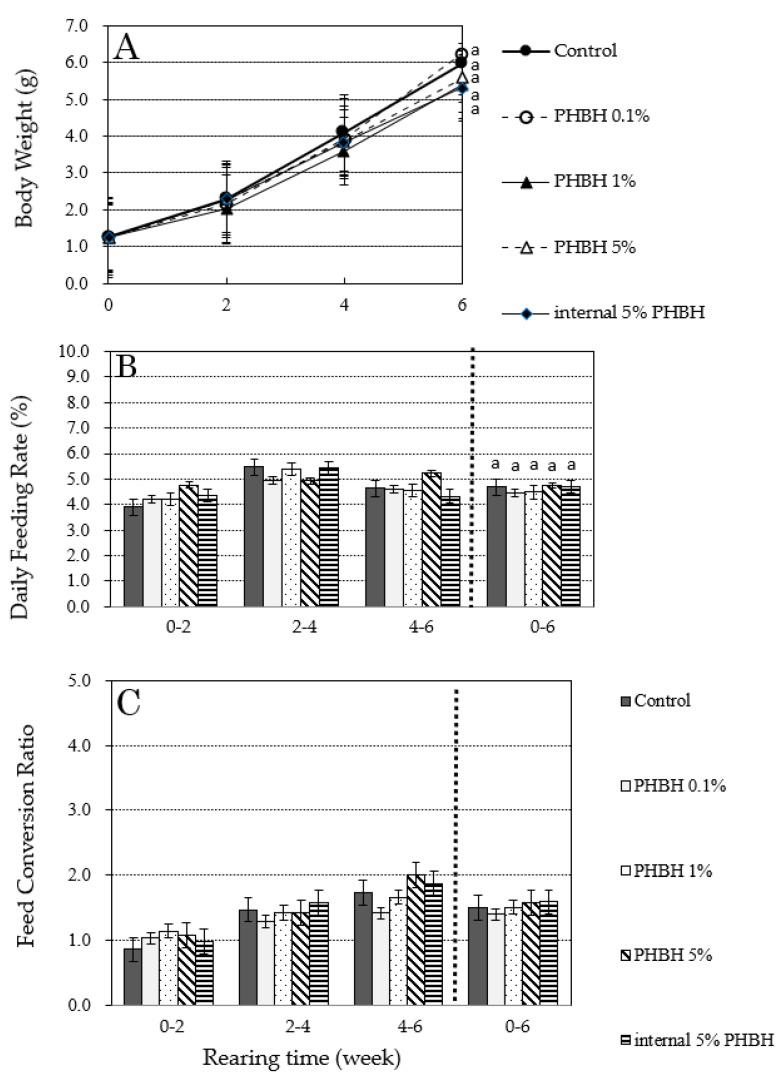
Increases in body weight (**A**) and changes in daily feeding rate (**B**) and feed conversion ratio (**C**) of *Marsupenaeus japonicus* during a 6-week feeding test with feed containing different amounts of poly-hydroxybutyrate-co-hydroxyhexanoate (PHBH). Different superscript letters show significant differences (*p* < 0.05).

Results of the experiment to evaluate the effect of adding PHBH to the diet on the growth of *M. japonicus* are shown in Figure 3. Increases in body weight (Figure 3A), change in daily feeding rate (Figure 3B), and change in feed conversion ratio (Figure 3C) are shown as average values of duplicate tests. Between weeks 4 and 6, there was a temporary interruption in the water supply to one of the experimental tanks (0.1% PHBH), and there were many dead shrimps due to oxygen depletion. In addition, some dead individuals were observed in each tank, including in the control, just before weight measurement at week 6. However, simple testing (see Section 2.6) revealed no pathogenic bacterial infection (data not shown). These dead individuals were eliminated from the calculation of the growth parameters.

At the end of the experimental period (i.e., on day 43), average body weights were 5.97 g in the control shrimp; 6.23 g, 5.40 g, and 5.58 g in the shrimp fed 0.1%, 1%, and 5% PHBH, respectively; and 5.31 g in the shrimp fed internal 5% PHBH (Figure 3A). The daily feeding rate for the experimental period was 4.68% in the control shrimp; 4.45%, 4.49%, and 4.74% in the shrimp fed 0.1%, 1%, and 5% PHBH, respectively; and 4.70% in the shrimp fed internal 5% PHBH (Figure 3B). Feed conversion ratios for the experimental period were 1.50 in the control shrimp; 1.40, 1.51, and 1.57 in the shrimp fed 0.1%, 1%, and 5% PHBH, respectively; and 1.59 in the shrimp fed internal 5% PHBH (Figure 3C). Final body weights, daily feeding rates, and feed conversion ratios for the experiential period were comparable (*p* > 0.05) between the control shrimp and those fed the feed containing PHBH, indicating that PHBH had no influence on the growth of *M. japonicus*.

### 3.4. In Vivo Bacterial Challenge Test

**Figure 4:**
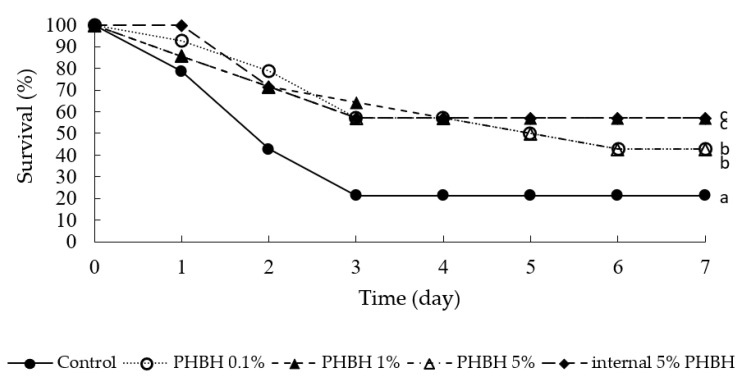
Survival rates of *Marsupenaeus japonicus* after infection with the shrimp-pathogenic bacterium *Vibrio penaeicida*. Nine weeks prior to bacterial infection and 1 week after infection, the shrimp were fed the feed containing different amounts of poly-hydroxybutyrate-co-hydroxyhexanoate (PHBH). Different superscript letters show significant differences (*p* < 0.05).

Figure 4 shows the survival rates of shrimps from the growth experiment in the week after being infected with *V. penaeicida*. In the shrimps reared on the standard diet (control), the survival rate was 78.6% on day 1, 42.9% on day 2, and 21.4% on days 3–7. In contrast, the survival rates of the shrimps reared on feed containing PHBH were significantly higher (≥50%) than those of the control shrimp. *Vibrio penaeicida* infection was detected in all dead, but none of surviving, shrimps (data not shown).

## 4. Discussion

Some previous studies indicated that adding organic acids/acidifiers (i.e., short-chain fatty acids) to the diet of aquaculture brought beneficial effects on the growth of shrimp and other aquatic animals [37,38]. Building on the findings of previous studies using water-soluble FAs and hydroxyalkanoic acids, the beneficial effects of PHB, a water-insoluble polymer of 3HB, on resistance to infection and growth were examined in aquatic animals [19,20,22,26,27,28,39]. However, high concentrations of 3HB, and consequently of PHB, were still needed before beneficial effects were observed [19,20,40]. In addition, it has been reported that short-chain FAs inhibit the growth of *Salmonella enteritidis* and *S. typhimurium* in a pH-dependent manner [41,42,43]. This pH dependence can be explained by the fact that fatty acids are able to pass through the cell membrane only in their undissociated form [44], which is the more abundant form at low pH.

In the present study, another bio-degradable hydroxyalkanoic acid polymer, PHBH, a polymer of 3HB and 3HH was used. Given that the number of carbons in 3HH (C = 6) is greater than that in 3HB (C = 4), it could be expected that PHBH would have the greater growth suppression activity in pathogenic *Vibrio* species. Here, we found that 3HH markedly inhibited the growth of the shrimp-pathogenic bacterium *V. penaeicida* in vitro (Figure 2C) and that the growth suppression activity of 3HH was much greater than that of 3HB at both pH 6.0 and pH 7.0 (Figure 2A,C,E,G). To the authors’ knowledge, this is the first report that 3HH has a notable inhibitory effect on the growth of a shrimp-pathogenic *Vibrio* species. These results suggest that we can expect a greater inhibitory effect of PHBH against pathogenic *Vibrio* bacteria compared with that of PHB, and that supplementation of standard shrimp diet with PHBH will improve shrimp aquaculture yields.

Moreover, the observed suppression of the growth of *V. penaeicida* by 3HB and 3HH at pH 6.0 were much greater than those at pH 7.0, indicating that these activities are pH dependent (Figure 2). In shrimp with a body length of around 8–12 cm (body weights about 6–19 g), the pH of the gut was within the range of 5.9–6.7 (average 6.29 ± SD 0.20) (Figure 1B). This may also be the first report on pH values of gut contents, and information that there was no relationship between pH value and body length of shrimps. Although results using pH test paper were not so accurate and had some error, results still suggest that the gut environment of the shrimp was appropriate for 3HB and 3HH to exert their inhibitory activates on the growth of *V. penaeicida*. Interestingly, the average pH of the gut of shrimp reared using feed containing 5% (*w*/*w*) PHBH was significantly lower than that of shrimp fed the standard diet alone (Figure 1A). Unfortunately, these results were obtained from only two individuals for each. For future studies, higher replication numbers should be included to verify these results. A similar decrease in intestinal pH has also been reported in European sea bass fed a diet containing PHB [36]. In a study of PHB, Defoirdt et al. showed that the concentration of 3HB in brine shrimp increased along with increasing dose of PHB in the rearing water [25]. In the present study, we did not directly determine the concentrations of 3HB and 3HH in the shrimp gut. However, because the pH of the gut of shrimp fed with a feed containing PHBH was lower than that in shrimp fed the standard diet (Figure 1A), it might be expected that PHBH is also degraded, at least partially, to its constituent monomers (3HB and 3HH) and oligomers in the shrimp gut.

Even if 3HB and 3HH produced by the degradation of PHBH in the shrimp gut inhibit the growth of pathogenic bacteria, PHBH would not be suitable as a dietary supplement if it has a negative effect on shrimp growth. We, therefore, examined the effect of PHBH on the growth of *M. japonicus*. Addition of PHBH to the standard diet increased total amount of “organic matter” for shrimps. Replacement of a part of standard diet by PHBH decreased the amount of nutrient. However, we found no significant differences in three growth parameters (i.e., increase in body weight, daily feeding rate, and feed conversion ratio) between shrimp reared on standard diet or on feed containing PHBH (0.1%, 1%, or 5% PHBH, or internal 5% PHB) (Figure 3). These results indicate that adding up to 5% (*w*/*w*) PHBH to the diet has no negative effects on shrimp growth for up to six weeks. Moreover, the results of our in vivo challenge test showed that the survival rates of shrimps reared on feed containing PHBH were significantly higher than those in shrimp reared on the standard diet (Figure 4). From these results, it is reasonable to conclude that 3HH and 3HB derived from degraded PHBH in the shrimp gut suppressed the growth of *V. penaeicida*.

## 5. Conclusions

In conclusion, our results indicate that supplementation of standard diet with PHBH increases the resistance of shrimps to infection by the shrimp-pathogenic bacterium *V. penaeicida*. The addition of PHBH to shrimp diet is expected to be useful for developing high-value-added feeds for aquaculture shrimp and consequently for increasing shrimp production yields.

## Figures and Tables

**Table 1:** Ingredient composition of the standard diet.

Chemical Composition	g/100g
Crude Protein	57.0
Crude Lipid	9.6
Fiber	3.0
Ash	16.0
Others	5.8
Water	8.6
Total	100

**Table 2:** Formulated compositions of the experimental feed.

Feed Component	PHBH %				Internal 5%
	Control	0.1	1	5	
Standard Diet	100	100	100	100	95
PHBH	0	0.1	1	5	5

## Data Availability

The data that support the findings of this study are available from the corresponding author [K. Fukami], on a reasonable request.

## References

[1] FAO (2020). The State of World Fisheries and Aquaculture 2020: Sustainability in Action.

[2] Aksornkoae S., Tokrisna R., Barbier E.B., Sathirathai S. (2004). Overview of shrimp farming and mangrove loss in Thailand. Shrimp Farming and Mangrove Loss in Thailand.

[3] Teeyaporn K., Fukami K., Songsangjinda P., Muangyao P. (2011). Isolation and characterization of *Noctiluca*-killing bacteria from a shrimp aquaculture pond in Thailand. Fish. Sci..

[4] Ferreira J.G., Falconer L., Kittiwanich J., Ross L., Saurel C., Wellman K., Zhu C.B., Suvanachai P. (2015). Analysis of production and environmental effects of Nile tilapia and white shrimp culture in Thailand. Aquaculture.

[5] Muangyao P., Fukami K., Songsangjinda P., Predalumpaburta Y. (2020). Stimulation by gutweed to increase the abundance of insect larvae as food for shrimp aquaculture in Thailand. Aquaculture.

[6] Momoyama K., Muroga K. (2005). Diseases of Cultured Kuruma Shrimp in Japan: A Review. Fish Pathol..

[7] Annual Changes in Production of Kuruma Shrimp (Marsupenaeus Japonicus) in Japan. https://ieben.net/data/catch/sea-farm/japan-tdfk/kurumaebi.html.

[8] Sakai T., Hirae T., Yuasa K., Kamaishi T., Matsuyama T., Miwa S., Oseko N., Iida T. (2007). Mass mortality of cultured kuruma prawn *Penaeus japonicus* caused by *Vibrio nigripulchritudo*.. Fish. Pathol..

[9] Mandal A., Das S.K. (2018). Comparative efficacy of neem (*Azadirachta indica*) and non-neem supplemented biofloc media in controlling the harmful luminescent bacteria in natural pond culture of *Litopenaeus vannaemei*.. Aquaculture.

[10] Thompson F.L., Iida T., Swings J. (2004). Biodiversity of vibrios. Microbiol. Mol. Biol. Rev..

[11] Austin B., Zhang X.H. (2006). *Vibrio harveyi*: A significant pathogen of marine vertebrates and invertebrates. Lett. Appl. Microbiol..

[12] Moriarty D.J.W., Bell C.R., Brylinsky M., Johnson-Green P. (1999). Disease control in shrimp aquaculture with probiotic bacteria. Microbial Biosystems: New Frontiers, Proceedings of the 8th International Symposium on Microbial Ecology, Halifax, NS, Canada, 1 August 1 1998.

[13] Decamp O., Moriarty D.J.W., Lavens P. (2008). Probiotics for shrimp larviculture: Review of field data from Asia and Latin America. Aquac. Res..

[14] Wang H., Wang C., Tang Y., Sun B., Huang J., Song X. (2018). *Pseudoalteromonas* probiotics as potential biocontrol agents improve the survival of *Penaeus vannamei* challenged with acute hepatopancreatic necrosis disease (AHPND)-causing *Vibrio parahaemolyticus*.. Aquaculture.

[15] Teo J.W.P., Tan T.M.C., Poh C.L. (2002). Genetic determinants of tetracycline resistance in *Vibrio harveyi*.. Antimicrob. Agents Chemother..

[16] Liu H., Wang Y., Gao J., Jiang H., Yao J., Gong G., Chen X., Xu W., He X. (2020). Antimicrobial activity and virulence attenuation of citral against the fish pathogen *Vibrio alginolyticus*.. Aquaculture.

[17] Defoirdt T., Boon N., Sorgeloos P., Verstraete W., Bossier P. (2009). Short-chain fatty acids and poly-β-hydroxyalkanoates: (new) biocontrol agents for a sustainable animal production. Biotech. Adv..

[18] Defoirdt T., Halet D., Sorgeloos P., Bossier P., Verstraete W. (2006). Short-chain fatty acids protect gnotobiotic *Artemia franciscana* from pathogenic *Vibrio campbellii*.. Aquaculture.

[19] Nhan D.T., Wille M., De Schryver P., Defoirdt T., Bossier P., Sorgeloos P. (2010). The effect of poly-β-hydroxybutyrate on larviculture of the giant freshwater shrimp *Macrobrachium rosenbergii*.. Aquaculture.

[20] Defoirdt T., Halet D., Vervaeren H., Boon N., Van De Wiele T., Sorgeloos P., Bossier P., Verstraete W. (2007). The bacterial storage compound poly-ß-hydroxybutyrate protects *Artemia franciscana*, from pathogenic *Vibrio campbellii*.. Environ. Microbiol..

[21] Suriyamongkol P., Weselake R., Narine S., Moloney M., Shah S. (2007). Biotechnological approaches for the production of polyhydroxyalkanoates in microorganisms and plants—A review. Biotechnol. Adv..

[22] Sui L., Cai J., Sun H., Wille M., Bossier P. (2012). Effect of poly-β-hydroxybutyrate on Chinese mitten crab, *Eriocheir sinensis*, larvae challenged with pathogenic *Vibrio anguillarum*.. J. Fish Dis..

[23] Leopoldo J., Laranja Q., Ludevese-Pascual G.L., Amar E.C., Sorgeloos P., Bossier P., De Schryver P. (2014). Poly-β-hydroxybutyrate (PHB) accumulating *Bacillus* spp. Improve the survival, growth and robustness of *Penaeus mondon* (Fabricius, 1798) postlarvae. Vet. Microbiol..

[24] Thai T.Q., Wille M., Garcia-Gonzalez L., Sorgeloos P., Bossier P., De Schryver P. (2014). Poly-ß-hydroxybutyrate content and dose of the bacterial carrier for *Artemia* enrichment determine the performance of giant freshwater shrimp larvae. Appl. Microbiol. Biotechnol..

[25] Hung N.V., De Schryver P., Tran T.T., Garcia-Gonzalez L., Bossier P., Nevejan N. (2015). Application of poly-β-hydroxybutyrate (PHB) in mussel larviculture. Aquaculture.

[26] Situmorang M.L., De Schryver P., Dierckens K., Bossier P. (2016). Effect of poly-β-hydroxybutyrate on growth and disease resistance of Nile tilapia *Oreochromis niloticus* juveniles. Vet. Microbiol..

[27] Gao M., Du D., Bo Z., Sui L. (2019). Poly-βhydroxybutyrate (PHB)-accumulating *Halomonas* improves the survival, growth, robustness and modifies the gut microbial composition of *Litopenaeus vannamei* postlarvae. Aquaculture.

[28] Defoirdt T., Anh N.T.M., De Schryver P. (2018). Virulence-inhibitory activity of the degradation product 3-hydroxybutyrate explains the protective effect of poly-β-hydroxybutyrate against the major aquaculture pathogen *Vibrio campbellii*.. Sci. Rep..

[29] Shimamura E., Kasuya K., Kobayashi G., Shiotani T., Shima Y., Doi Y. (1994). Physical Properties and Biodegradability of Microbial Poly (3-hydroxybutyrate-co-3-hydroxyhexanoate). Macromolecules.

[30] Doi Y., Kitamura S., Abe H. (1995). Microbial Synthesis and Characterization of Poly(3-hydroxybutyrate-co-3-hydroxyhexanoate). Macromolecules.

[31] Fujiki T. (2013). Biodegradable plastic production by microorganism. Microbiol. Cult. Coll..

[32] Arikawa H., Matsumoto K. (2016). Evaluation of gene expression cassettes and production of poly (3-hydroxybutyrate-co-3-hydroxyhexanoate) with a fine modulated monomer composition by using it in *Cupriavidus necator*.. Microb. Cell Fact..

[33] Immanuel G., Sivagnanavelmurugan M., Palavesam A. (2012). Antibacterial effect of short-chain fatty acids on gnotobiotic *Artemia franciscana* nauplii against *Vibrio parahaemolyticus*.. Aquac. Res..

[34] Hung N.V., Bossier P., Hong N.T.X., Ludeseve C., Garcia-Gonzalez L., Nevejan N., De Schryver P. (2019). Does *Ralstonia eutropha*, rich in poly-β hydroxybutyrate (PHB), protect blue mussel larvae against pathogenic *vibrios*?. J. Fish Dis..

[35] Fukami K., Nishijima T., Hata Y. (1992). Availability of deep seawater and effects of bacteria isolated from deep seawater on the mass culture of food microalga *Chaetoceros ceratosporum*.. Nippon Suisan Gakkaishi.

[36] Ali M.F.Z., Yasin I.A., Ohta T., Hashizume A., Ido A., Takahashi T., Miura C., Miura T. (2018). The silkrose of *Bombyx mori* effectively prevents vibriosis in penaeid prawns via the activation of innate immunity. Sci. Rep..

[37] Mine S., Boopathy R. (2011). Effect of organic acids on shrimp pathogen, Vibrio harveyi. Curr. Microbiol..

[38] Hoseinifar S.H., Sun Y.-Z., Caipang C.M. (2017). Short-chain fatty acids as feed supplements for sustainable aquaculture: An updated view. Aquacul. Res..

[39] De Schryver P., Shima A.K., Kunwar P.S., Baruah K., Verstraete W., Boon N., Boeck G., Bossier P. (2010). Poly-β-hydroxybutyrate (PHB) increases growth performance and intestinal bacterial range-weighted richness in juvenile European sea bass, *Dicentrarchs labrax*.. Appl. Microbiol. Biotechnol..

[40] Halet D., Defoirdt T., Van Damme P., Vervaeren H., Forrez I., Van de Wiele T., Boon N., Sorgeloos P., Bossier P., Verstraete W. (2007). Poly-ß-hydroxybutyrate-accumulating bacteria protect gnotobiotic *Artemia franciscana* from pathogenic *Vibrio campbellii*.. FEMS Microbiol. Ecol..

[41] McHan F., Shotts E.B. (1993). Effects of short-chain fatty acids on the growth of Salmonella tyrhimurium in an in vitro system. Avian Dis..

[42] Durant J.A., Lowry V.K., Nisbet D.J., Stanker L.H., Corrier D.E., Ricke S.C. (2000). Late logarithmic *Salmonella typhimurium* HEp-2cell association and invasion response to short-chain fatty acid addition. J. Food Saf..

[43] Van Immerseel F., De Buck J., Pasmans F., Velge P., Botteau E., Fievez V., Haesebrouck F., Ducatelle R. (2003). Invasion of *Salmonella enteritidis* in avian intestinal epithelial cells in vitro is influenced by short-chain fatty acids. Int. J. Food Microbiol..

[44] Sun C.Q., O’ Connor C.J., Turner S.J., Lewis G.D., Stanley R.A., Roberton A.M. (1998). The effect of pH on the inhibition of bacterial growth by physiological concentrations of butyric acid: Implications for neonates fed on suckled milk. Chem. Biol. Interact..

